# Lavender oil suppresses indoleamine 2,3-dioxygenase activity in human PBMC

**DOI:** 10.1186/1472-6882-14-503

**Published:** 2014-12-16

**Authors:** Johanna M Gostner, Markus Ganzera, Kathrin Becker, Simon Geisler, Sebastian Schroecksnadel, Florian Überall, Harald Schennach, Dietmar Fuchs

**Affiliations:** Division of Medical Biochemistry, Biocenter, Medical University of Innsbruck, Innsbruck, Austria; Institute of Pharmacy, Pharmacognosy, University of Innsbruck, Innsbruck, Austria; Division of Biological Chemistry, Biocenter, Medical University of Innsbruck, Innsbruck, Austria; Central Institute of Blood Transfusion and Immunology, University Hospital, Innsbruck, Austria

**Keywords:** Lavender oil, Tryptophan, Indoleamine 2,3-dioxygenase, Neopterin, Kynurenine

## Abstract

**Background:**

Lavender remedies have been used in traditional medicine because of antimicrobial, anti-inflammatory and mood alleviating effects, but underlying molecular mechanisms are not yet fully elucidated. Recently, studies investigating the effects of lavender oil in the context of psychiatric disorders have indicated potent pharmacological properties. Metabolism of tryptophan by indoleamine 2,3-dioxygenase (IDO) was found to provide a biochemical link between immunology and neuroendocrinology and to be a frequent target of natural products.

**Methods:**

In this *in vitro* study, interferences of lavender oil and constituents (-)-linalool, (+)-α-pinene and (+)-limonene with tryptophan catabolism by IDO and formation of neopterin via guanosine triphosphate (GTP)-cyclohydrolase-I and of interferon-γ have been investigated using unstimulated and phytohemagglutinin (PHA)-stimulated human peripheral blood mononuclear cells (PBMC).

**Results:**

Treatment with lavender oil dose-dependently suppressed PHA-induced tryptophan breakdown and kynurenine formation. Similar effects were observed for the three constituents. In parallel, formation of neopterin and interferon-γ was diminished upon lavender oil treatment. In unstimulated PBMC, effect of lavender oil treatment was similar, but less pronounced.

**Conclusion:**

Data from this *in vitro* study suggest that lavender oil treatment might contribute to the modulation of the immune and neuroendocrine system by interfering with activation-induced tryptophan breakdown and IDO activity.

## Background

Lavender (*Lavandula angustifolia*) essential oil consists of a mixture of mono- and sesquiterpenoid alcohols, esters, oxides and ketones, containing linalool, linalyl acetate, 1,8-cineole, terpinen-4-ol, β-caryophyllene and camphor as primary components [1, 2].

Lavender and its essential oil have been used since centuries due to antiseptic, antimicrobial and sedative effects. In today’s folk and complementary medicine, the oil is applied also for the treatment of conditions such as anxiety, restlessness, insomnia and depression. Administration routes include absorption via the respiratory tract (aromatherapy) or oral ingestion [3]. Although there is evidence-based information on the pharmaceutical efficacy of lavender oil for the treatment of anxiety-related disturbances [4], its therapeutic significance was little appreciated for a long time, due to the lack of larger clinical trials, but also due to methodological problems in constituent identification and standardization of such complex multicomponent preparations.

Recently, Kasper *et al*. demonstrated the therapeutic efficacy of the lavender oil preparation Silexan for the treatment of subsyndromal anxiety disorder in a randomized, double-blind, placebo controlled trial [5]. Lavender oil treatment was found to alleviate anxiety related symptoms such as restlessness, disturbed sleep as well as somatic complaints, whereby the product demonstrated good tolerability without provoking greater adverse effects [6].

However, only few reports on the specific neurobiochemical actions of lavender oil are available. Best discussed in the literature are the anxiolytic, analgesic and anti-inflammatory effects of one of the oil’s principal components, linalool and its derivatives like linalyl acetate [7]. The antinociceptive potential of linalool was studied in several animal studies, e.g. it has been shown to interfere with glutamatergic transmission in mice [8, 9] and to modify the nicotinic receptor-ion channel kinetics at the neuromuscular junction [10].

Importantly, immune activation and inflammation are strongly associated with an increase of mood disorders [11, 12]. Several biochemical links between psychoneuroimmunology and neuropsychopharmacology have been dissected in the past. The catabolism of the essential amino acid tryptophan, known for its essential role in antimicrobial defence, has turned out as an important link between the immunological network and neuroendocrine functions [12, 13]. The enzyme indoleamine 2,3-dioxygenase (IDO, EC 1.13.11.52) catalyses the rate-limiting step in the conversion of tryptophan to kynurenine and becomes highly activated in macrophages and is induced also in many other cell types upon exposure to pro-inflammatory cytokine interferon-γ (IFN-γ) signaling in the course of the cellular immune response [12]. Tryptophan depletion creates an anti-proliferative environment against target cells and contributes to the antimicrobial effects of activated macrophages [14]. However, a reduction in plasma tryptophan leads in consequence to low serotonin (5-HT) synthesis and further, several tryptophan breakdown products are known to exert neuroactive effects [11].

During the cellular immune response, in parallel to IDO, guanosine triphosphate (GTP)-cyclohydrolase-I (GTP-CH-I, EC 3.5.4.16) is induced by IFN-γ. GTP-CH-I is the key enzyme in the biosynthesis of neopterin, a marker molecule for immune system activation [15, 16]. As tryptophan metabolism may occur not only via IDO but also via hepatic tryptophan 2,3-dioxygenase (TDO), a concomitant determination of immune activation marker neopterin is suitable to judge the contribution of inflammation in changes of tryptophan levels [12]. Neopterin levels and kynurenine to tryptophan ratio (Kyn/Trp) have turned out as useful markers for a variety of diseases that are associated with chronic immune activation such as infections, autoimmune syndromes, malignancies or neurodegeneration [12, 16].

The aim of this study was to evaluate the effects of lavender essential oil and some of its constituents on tryptophan catabolism, by using the well established model system of freshly isolated human peripheral blood mononuclear cells (PBMC), stimulated or not with the mitogen phytohemagglutinin (PHA) [17]. Determination of Kyn/Trp and neopterin levels in cell culture supernatants is used as sensitive and reliable read-out for the activation status of PBMCs. The terpene alcohol (-)-linalool, a major constituent of lavender oil that is also contained in several edible plant species, as well as two minor lavender oil constituents (+)-α-pinene and (+)-limonene were chosen as reference compounds for analysis, due to their reported anti-inflammatory properties [18, 19].

## Methods

### Chemicals

Phytohemagglutinin (PHA), (+)-α-pinene, (-)-linalool and (+)-limonene were purchased from Sigma Aldrich (Vienna, Austria). Freshly prepared lavender oil (Aetheroleum Lavanduli) was purchased from a local pharmacy (Tiroler Adler Apotheke, Innsbruck, Austria).

### Analytical methods

Analyses of lavender oil were performed on a Perkin-Elmer Autosystem gas chromatograph (Norwalk, USA) equipped with FID, split-splitless injector and a Permabond SE-54-DF capillary column (50 m x 0.32 ID; 0.25 μm film thickness) from Macherey-Nagel (Düren, Germany). Helium (1 mL/min) was used as carrier gas, and injector and detector temperatures were set to 220 and 240°C, respectively. The injected sample volume was 0.5 μL with a split ratio of 1:4. The following temperature gradient was applied for all separations: for the first 10 min isotherm at 85°C, then the temperature was increased to 180°C (10°C/min), to keep this setting for 5.5 min. For GC analysis, 60.0 mg of essential oil was dissolved in 5.00 mL dichloromethane (Chromasolv; Riedel-de-Haen, Seelze, Germany). For preparation of the standard solutions 30.0 mg of each compound ((+)-α-pinene, (-)-linalool and (+)-limonene) was placed in one 5.00 mL volumetric flask, which was filled up to volume with DCM (level 1). Further concentration levels were prepared by serial dilution using the same solvent, so that the covered linear range was from 6.0 to 0.08 mg/mL for all compounds. External calibration curves were constructed for the three available reference compounds, and the following regression equations were obtained: y = 14429 x – 336.59 ((+)-α-pinene), y = 16024 x – 902.95 ((+)-limonene) and y = 15780 x – 1407.70 ((-)-linalool). The respective correlation coefficients were 0.9997 or higher.

### Isolation of human PBMC

The study was performed in accordance with the Helsinki declaration. PBMC were isolated from whole blood obtained from healthy donors of whom informed consent was obtained that their donated blood might be used for scientific purposes in case when it was not selected for transfusion. The local ethics committee confirmed that no further approval is required for using anonymized leftover specimens from blood donations of the local blood bank for scientific purposes. Separation of blood cells was performed by density centrifugation (Lymphoprep, Nycomed Pharma AS, Oslo, Norway) as described [17, 20]. After isolation, PBMC were washed three times in phosphate buffered saline containing 1 mM EDTA. Cells were cultivated in RPMI 1640 supplemented with 10% heat-inactivated fetal calf serum (Biochrom, Berlin, Germany), 2 mM L-glutamine (Serva, Heidelberg, Germany) and 50 μg/mL gentamicin (Bio-Whittaker, Walkersville, MD) at 37°C in a humidified atmosphere containing 5% CO_2_.

### Cell treatment

In brief, isolated PBMC were seeded at a density of 1.5 × 10^6^ cells/mL in supplemented RPMI 1640 medium and pre-incubated for 30 min with or without different concentrations of lavender oil [0.1 – 5%] or constituents ((+)-α-pinene [31.25 – 250 μM], (-)-linalool [25 – 1000 μM] and (+)-limonene [50 – 250 μM]). Then, cells were stimulated or not with 10 μg/mL PHA for 48 h. For each of the experiments run in duplicates, PBMC were prepared freshly from blood of at least three different healthy donors [20].

### Cell viability

CellTiter-Blue assay (Promega, Germany) was used to determine cell viability. The half maximal (50% inhibitory) concentration (IC_50_) was calculated by using the CalcuSyn software (Biosoft, UK) [21].

### Measurement of tryptophan, kynurenine, neopterin and IFN-γ concentrations

After 48 h of incubation, the accumulated tryptophan breakdown and neopterin formation reach a plateau [17, 20]. PBMC supernatants were harvested by centrifugation. Tryptophan and kynurenine concentrations were measured by high performance liquid chromatography (HPLC) using 3-nitro-L-tyrosine as an internal standard [22]. To estimate the activity of IDO, Kyn/Trp was calculated and expressed as μmol kynurenine/mmol tryptophan. Neopterin and IFN-γ concentrations were measured by ELISA (BRAHMS, Hennigsdorf/Berlin, Germany and R&D, Biomedica, Vienna, Austria) according to the manufacturers’ instructions with a detection limit of 2 nmol/L neopterin and 8 pg/mL IFN-γ. Due to limited sample volumes, IFN-γ measurements were performed in a smaller set of 32 lavender oil treated samples only.

### Statistical analysis

Data were analysed by using the Statistical Package for the Social Sciences (version 19, SPSS, Chicago, III, USA). To take into account that not all collected data followed a normal distribution, non-parametric Friedman and Wilcoxon signed-rank test were applied. P-values below 0.05 were considered to indicate significant differences.

## Results

### GC analysis

**Figure 1:**
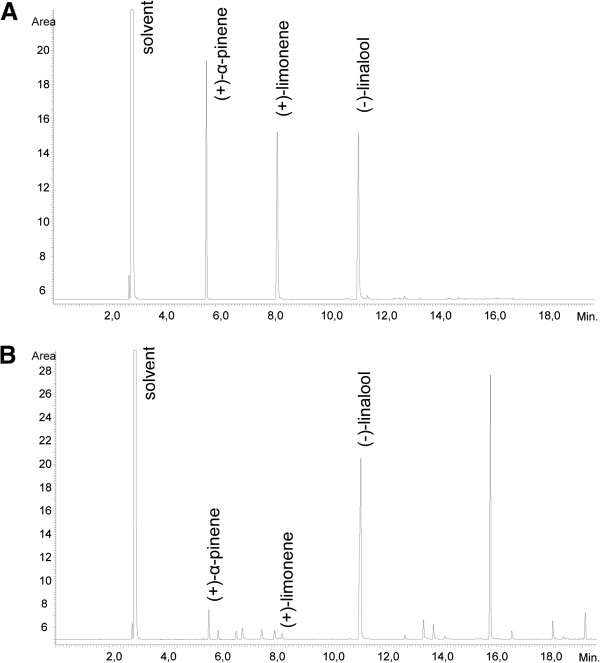
**Determination of (+)-α-pinene, (-)-linalool and (+)-limonene in lavender oil.** GC analysis of a standard mixture **(A)** and lavender essential oil **(B)**.

The three compounds (+)-α-pinene, (-)-linalool and (+)-limonene could be separated with GC and assigned in lavender essential oil (Figure 1). The respective retention times were found to be 5.72 min ((+)-α-pinene), 8.34 min ((+)-limonene) and 11.38 min ((-)-linalool). Their content in the essential oil sample was 3.70% (σ_rel_ = 0.29; n = 3) for (+)-α-pinene, 1.29% (σ_rel_ = 0.11) for (+)-limonene, and 33.09% (σ_rel_ = 0.64) for (-)-linalool.

### Effect of lavender oil and its constituents on cell viability

**Figure 2:**
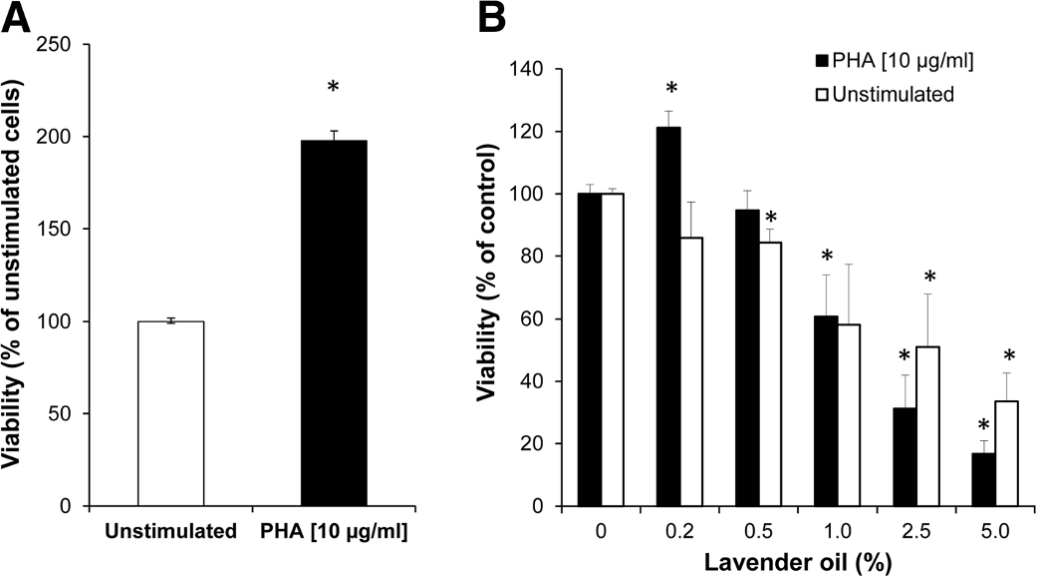
**Effect of lavender oil on cell viability. (A)** Viability of PBMC cells after treatment with (black bars) or without PHA (white bars) (*P < 0.05, compared to unstimulated control). **(B)** Viability of unstimulated (white bars) and PHA-stimulated (black bars) PBMC after incubation with increasing concentrations of lavender oil for 48 h, expressed as % of baseline (*P < 0.05, compared to cells without added lavender oil). Results shown are the mean values ± SEM of two independent experiments run in triplicates.

Cytotoxicity was evaluated after 48 h incubation with lavender oil in a concentration range from 0.2 to 5.0% in unstimulated and PHA-stimulated PBMC. Treatment with the mitogen PHA [10 μg/mL] resulted in a significant increase of cell number due to the stimulated cell growth (Figure 2A). Reduced viability in the presence of lavender oil was measured with 0.5% addition in unstimulated and with 1.0% in PHA-stimulated cells, indicating the beginning of cytotoxic effects for higher treatment concentrations (Figure 2B). Viability decreased in a dose dependent manner, with IC_50_ values of 2.29% in unstimulated and 1.79% in stimulated cells.

No cytotoxic effects in the tested concentration ranges could be observed for (-)-linalool (IC_50_ ≥ 1000 μM) and (+)-limonene (IC_50_ ≥ 250 μM), while for (+)-α-pinene IC_50_ values of 195.41 μM in unstimulated PBMC and 127.36 μM in PHA-stimulated PBMC were determined.

### Effect of lavender oil and its constituents on tryptophan breakdown

**Figure 3:**
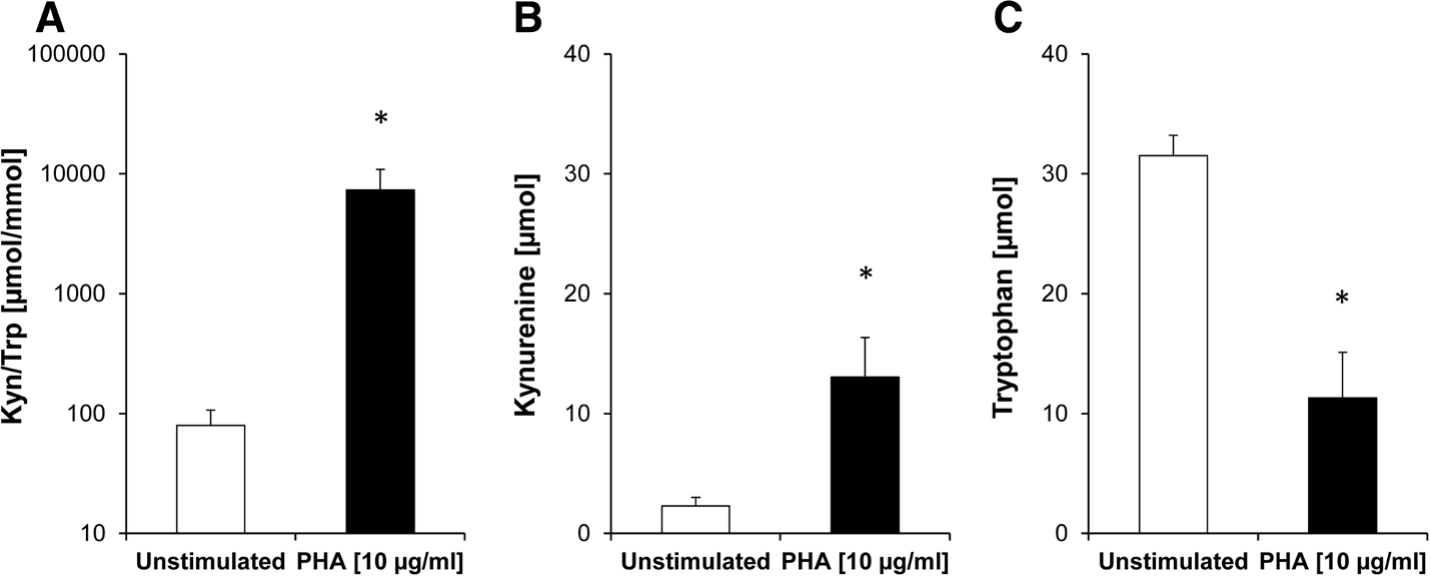
**Effect of PHA stimulation in PBMCs.** Indoleamine 2,3-dioxygenase activity, expressed as the kynurenine to tryptophan ratio (Kyn/Trp in μmol/mmol, note log scale) **(A)** and concentrations of kynurenine **(B)** and tryptophan **(C)** in unstimulated (white bars) and PHA-stimulated (black bars) PBMC. Results shown are the mean values ± SEM of four independent experiments run in duplicates (*P < 0.05, compared to unstimulated cells).

**Figure 4:**
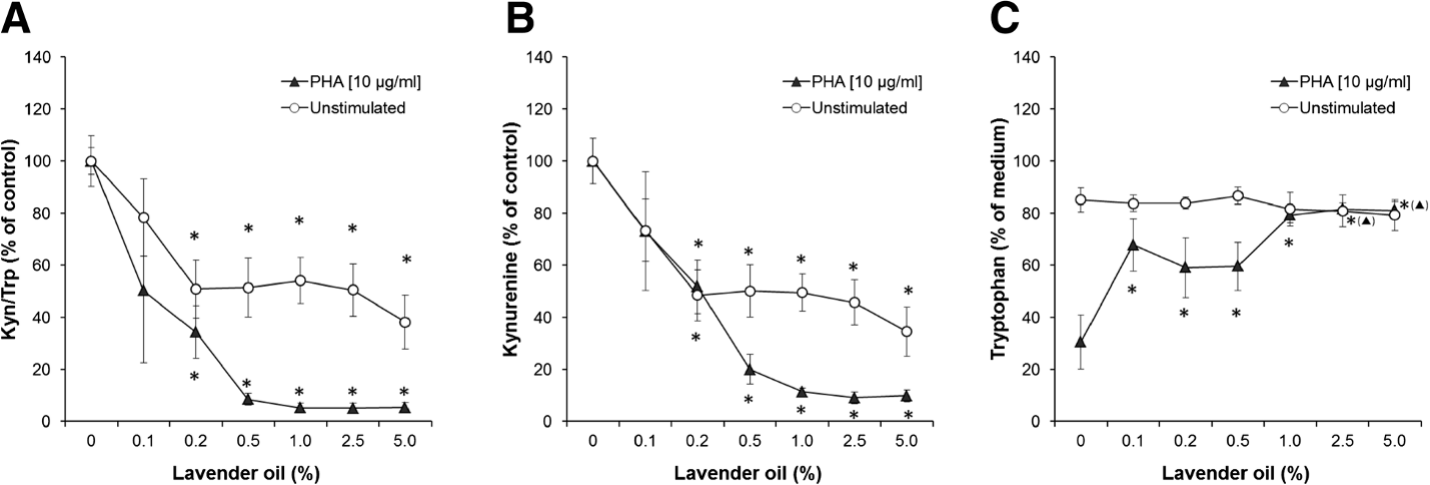
**Effect of lavender oil on tryptophan metabolism.** Effect of lavender oil on the kynurenine to tryptophan ratio (Kyn/Trp; **A)**, kynurenine **(B)** and tryptophan **(C)** concentrations in unstimulated (white circles) and PHA-stimulated PBMC (black triangles), expressed as % of baseline (control cells treated with or without PHA). Tryptophan concentrations are expressed as % of medium control. Results shown are the mean values ± SEM of four independent experiments run in duplicates (*P < 0.05, compared to baseline).

After an incubation period of 48 h, the average tryptophan concentration in supernatants of unstimulated PBMC was 31.5 ± 1.7 μmol/L (mean ± SEM; 85.2 ± 4.7% of initial medium content) and the mean kynurenine concentration was 2.3 ± 0.7 μmol/L, resulting in Kyn/Trp of 79.0 ± 28.0 μmol/mmol (Figure 3). Upon stimulation of cells with PHA, tryptophan levels decreased to 11.3 ± 3.8 μmol/L, while the mean kynurenine concentration increased to 13.1 ± 3.3 μmol/L. The Kyn/Trp was increased in PHA-treated samples approximately 100-fold in comparison to the unstimulated supernatants (all P < 0.05).By addition of lavender oil [0.1 – 5.0%] to unstimulated PBMC, no change of tryptophan concentrations but a significant decline of kynurenine and Kyn/Trp levels was apparent with the treatment concentrations of 0.2 to 5.0% (Figure 4). In PHA-stimulated PBMC, lavender oil suppressed tryptophan breakdown significantly at all treatment concentrations (0.1 to 5.0%), Kyn/Trp and kynurenine levels dropped dose-dependently and significantly (Figure 4). Addition of 1.0% of lavender oil completely inhibited tryptophan breakdown and restored tryptophan levels comparable to the concentration in unstimulated cell supernatants (79.4 ± 3.0% of medium content).

**Figure 5:**
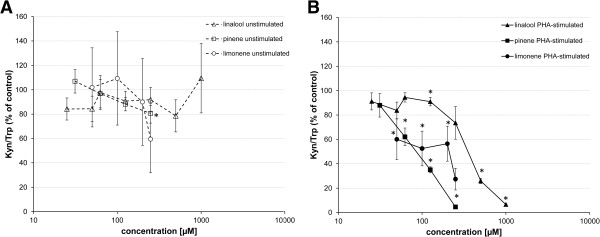
**Effect of (+)-α-pinene, (-)-linalool and (+)-limonene on tryptophan metabolism.** Effect of the lavender oil constituents on the kynurenine to tryptophan ratio (Kyn/Trp) in **(A)** unstimulated (white symbols) and **(B)** PHA-stimulated PBMC (black symbols), expressed as % of baseline (control cells treated with or without PHA). Results shown are the mean values ± SEM of three independent experiments run in duplicates (*P < 0.05, compared to baseline).

While in PHA-treated cells, the incubation with 0.5% of lavender oil resulted in a suppression of kynurenine to 19.9 ± 5.7% of PHA control, the same concentration decreased kynurenine levels only to 50.2 ± 10.1% in unstimulated supernatants (compared to unstimulated control). The effect of lavender oil on IDO activity as indicated by Kyn/Trp was also significantly reduced in both, mitogen-stimulated and unstimulated PBMC. Likewise, this effect was more striking in PHA-stimulated cells, where 0.5% of lavender oil addition resulted in a reduction of IDO activity to 8.5 ± 2.2% compared to the PHA control. The same lavender concentration decreased Kyn/Trp only to 51.4 ± 11.3% in unstimulated PBMC (compared to unstimulated controls). Overall, lavender oil induced effects were more pronounced in PHA-stimulated cells than in unstimulated ones.Neither (-)-linalool treatment (up to 1000 μM) nor (+)-limonene addition (up to 250 μM) resulted in a significant change of the Kyn/Trp ratio in unstimulated PBMC (Figure 5A). (+)-α-pinene did not affect IDO activity up to a concentration of 125 μM. Treatment of unstimulated PBMC with (+)-α-pinene at already cytotoxic concentrations (250 μM) resulted in a Kyn/Trp decrease of 19.4 ± 8.0% (Figure 5A). In PHA-stimulated cells, all constituents were able to strongly and significantly inhibit IDO activity in a dose-dependent manner at non-toxic concentrations (Figure 5B). With (+)-limonene and (+)-α-pinene, lower treatment concentrations led to a decrease of the Kyn/Trp ratio in stimulated cells (25 μM and 62.5 μM, respectively) than with (-)-linalool, were the inhibitory effect started with 125 μM.

### Effect of lavender oil on neopterin and IFN-γ production

**Figure 6:**
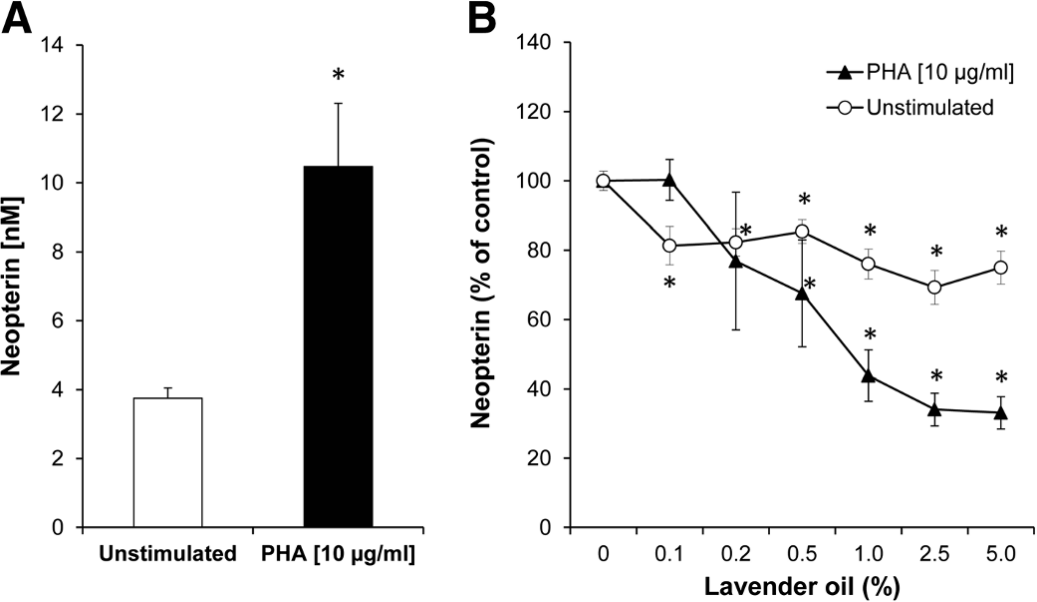
**Effect of lavender oil on neopterin production. (A)** Concentration of neopterin in unstimulated (white bar) and PHA-stimulated PBMC (black bar) (*P < 0.05, compared to unstimulated control). **(B)** Neopterin concentrations expressed as % of baseline (control cells treated with or without PHA) in the supernatants of unstimulated (white circles) and PHA-stimulated PBMC (black triangles), incubated with increasing concentrations of lavender oil for 48 h. Results shown are the mean values ± SEM of four independent experiments run in duplicates (*P < 0.05, compared to baseline).

After 48 h, neopterin concentrations were significantly higher (10.5 ± 1.8 nmol/L) in culture supernatant of PHA-stimulated cells than in supernatants of unstimulated PBMC (3.8 ± 0.3 nmol/L) (p < 0.05; Figure 6A). Also, IFN-γ levels were increased in mitogen stimulated cells (209.7 ± 73.4 pg/mL) in comparison to untreated cells (19.3 ± 14.5 pg/mL) (P < 0.05).The incubation of unstimulated PBMC with lavender oil resulted in the reduction of neopterin levels at all treatment concentrations (Figure 6B). At a concentration of 0.5% lavender oil and higher, neopterin formation was maximally decreased to 85.4 ± 3.4% in comparison to the untreated control cells. In PHA-stimulated cells, neopterin concentrations were significantly and dose-dependently lowered in the concentration range from 0.5 - 5.0% of lavender oil, with a reduction to 67.6 ± 15.5% at 0.5% lavender oil treatment, in comparison to the PHA-treated control.

IFN-γ concentrations were not affected in unstimulated PBMC upon lavender oil treatment, while in stimulated cells, IFN-γ levels decreased in a dose-dependent manner (data not shown). Addition of 0.5% of lavender oil resulted in a reduction of IFN-γ production until baseline levels of unstimulated cells (22.4 ± 5.9 pg/mL).

## Discussion

Anti-inflammatory properties of lavender oil and its constituents have been reported in several *in vitro* and *in vivo* studies. Lavender essential oil and constituents have been shown to interfere with key immunological pathways, e.g. nuclear factor kappa B (NF-κB) and p38 mitogen-activated protein kinase (MAPK) signaling as well as cytokine secretion [19, 23]. E.g., (+)-α-pinene, (-)-linalool and (+)-limonene were able to decrease interleukin-2 (IL-2) secretion and to increase the IL-10/IL-2 ratio in mouse primary splenocytes, which indicates their property to repress Th1 immune activation and suggest a potential inclination towards Th2 [19]. Furthermore, (-)-linalool was able to attenuate the production of lipopolysaccharide (LPS)-induced tumor necrosis factor α (TNFα) and IL-6 both in RAW 264.7 macrophages and in mice, and has been discussed as potential anti-inflammatory agent for preventing lung injury [19, 23].

The impact of the reference substances in attenuating Th1 immune response agrees with results of our study, which showed that non-toxic concentrations of (+)-α-pinene, (-)-linalool and (+)-limonene were able to inhibit mitogen-stimulated IDO activity in a model system of freshly isolated PBMC.

Also, lavender oil treatment was able to dose-dependently inhibit both tryptophan breakdown and kynurenine formation in supernatants of mitogen-stimulated PBMC. This inhibitory effect could already be detected at lavender oil concentrations that affected cell viability only slightly (0.1 to 0.5%). At higher treatment concentrations, effects on tryptophan and kynurenine were even stronger, however also cytotoxic effects of lavender oil increased. Interestingly, it has been shown that kynurenine metabolites are able to induce Th1 cell apoptosis [24]. Thus, we suggest that at low concentrations, lavender oil might beneficially influence cell viability by counteracting pro-apoptotic signaling, while at higher concentrations toxicity effects become prevalent. In studies with several compounds in the PBMC model [17, 20], IDO inhibition preceded substance toxicity phenomena, thus probably being a more sensitive indicator of cell death. Of note, the viability assay used in this study is based on the reduction of resazurin to fluorescent resorufin. Increased conversion rates may also indicate enhanced metabolic activity of cells, which does not always correlate with an increase in proliferation [25, 26].

Importantly, in mitogen-stimulated cells, a suppressive effect of lavender oil treatment on neopterin and IFN-γ concentrations could be observed. In unstimulated cells, lavender oil treatment had no influence on tryptophan and IFN-γ levels, but the formation of kynurenine and neopterin was suppressed to some extent. As PBMC were preincubated with the lavender oil before PHA addition, we suggest that the oil interferes mainly with IDO and GTP-CH-I stimulation. A basal activity of both enzymes is suggested to be present also in unstimulated cells, probably initiated due to the preceding cell isolation procedure. In unstimulated PBMC, tryptophan levels remained unaffected upon lavender oil treatment, e.g. with a 0.5% oil addition, 86.8 ± 3.2% of the initial medium content of tryptophan, corresponding to ~ 32 μmol/L, was still detectable after 48 h, while a significant reduction of kynurenine levels was observed. For 0.5% lavender oil treatment, kynurenine levels were reduced to 50.2 ± 10.1% compared to the untreated control, which corresponds to a reduction from 2.3 ± 0.7 μmol/L to 0.8 ± 0.1 μmol/L.

Of note, changes in immune parameters, such as impaired activities of immunocompetent cells, and involvement of inflammatory mediators and pro-inflammatory cytokines have been reported to be associated with behavioural alterations by several studies, and cell-mediated immune activation is suggested to be an important factor in distinct mental disturbances [11]. Behavioural changes can be induced by altered cytokine levels, e.g. studies of IFN-α treated patients showed therapy-induced depressive symptoms associated with activation of neuroendocrine pathways and altered serotonin metabolism [11, 27]. Within the cellular immune response, pro-inflammatory pathways are strongly induced, including neopterin production via GTP-CH-I and tryptophan catabolism via IDO, and the concentrations of these biomarkers have been found to be altered in mental disorders or diseases associated mood disturbances [12]. Enhanced neopterin concentrations together with low serum levels of tryptophan caused by increased tryptophan breakdown were shown to correlate with neuropsychiatric abnormalities like cognitive decline and depressive symptoms especially in long-lasting and chronic diseases [28].

Beside the important role of tryptophan catabolism in the regulation of inflammatory responses [29], tryptophan is a source for the production of 5-hydroxytryptophan, an intermediate in the biosynthesis of neurotransmitter serotonin. In states of persistent immune activation, availability of free serum tryptophan is diminished and as a consequence of reduced serotonin production, serotonergic functions may as well be affected [12].

About 95% of the body’s serotonin resides in the gut [30]. Furthermore, the gastrointestinal tract is rich in lymphocytes. Lavender oil treatment concentrations used for this *in vitro* study may appear relatively high, however *in vivo*, initial effects on IDO are suggested to be initiated already in the gastrointestinal tract, were such concentrations can be readily reached. In the study of Kasper et al., a treatment concentration of 80 mg/day was able to induce clinically meaningful and statistically significant anxiolytic effects [5].

Of note, deciphering specific bioactivities of isolated essential oil components is challenging because of the great number of constituents with similar physicochemical properties (e.g. lipophilic, high vapor pressure). In general, the major components reflect quite well the features of the essential oils from which they derive [31]. However minor constituent may contribute to the overall activity profile by modulating these activities and synergisms can play a major role [32]. (-)-Linalool is the most studied monoterpene regarding analgesic effects, a more detailed elucidation of its impact on the GABAergic system would help to dissect molecular details on its anticonvulsant, analgesic as well as anxiolytic activities [33]. For both (+)-limonene [34] and (+)-α-pinene [35] antinociceptive effects have been reported, however these effects are suggested to be strongly associated with their anti-inflammatory activities [33].

The here reported effect on tryptophan breakdown is not a unique property of lavender oil or the analysed constituents. In earlier studies using the identical cell-biological assay, similar effects on tryptophan metabolism have been found by investigating *Hypericum perforatum* extracts as well as Δ9-tetrahydrocannabinol and cannabidiol, indicating that the suppression of tryptophan breakdown and neopterin production might be an important but a more general aspect in the action of psychoactive compounds [36, 37]. Thus, although our findings are from *in vitro* experiments only, they might be relevant also for the *in vivo* situation.

Furthermore, kynurenine derivatives such as kynurenic and quinolinic acid and 3-hydroxykynurenine are known to be neuroactive and their hyper- or hypofunction is associated with neurological disorders and psychiatric diseases such as depression and schizophrenia [38]. The quinolinic acid to kynurenic acid ratio in the brain is discussed as a potential measure for conditions linked to excitotoxicity. Although both substances must be synthesized locally, because they are not able to cross the blood–brain barrier, other kynurenine pathway components such as tryptophan, kynurenine and 3-hydroxykynurenine can enter the brain, thus establishing a link between peripheral inflammation and brain tryptophan metabolism [39]. Additionally, also microglial cells and blood-borne cells within the brain can be stimulated to activate the kynurenine pathway in states of peripheral immune activation [38].

Scientific reports on the impact of IDO activity for different pathological conditions, including neuropsychiatric disturbances, account for IDO as a potential key pharmacological target. Several IDO inhibitors have been identified yet, and much effort will be necessary to evaluate their *in vivo* efficacy. The most prominent IDO inhibitor1-methyl tryptophan (1-MT) has been shown to counteract microbial-induced depressive-like symptoms in animal studies [40]. Beside the synthetic design of IDO antagonists via rational design strategies, also a variety of endogenous and exogenous antioxidants, such as vitamins, food supplements or preservatives, have been shown to suppress tryptophan catabolism in cellular model systems [17, 20]. Thereby, the modulation of tryptophan metabolism is suggested to be due to the interference of the test substances mostly with immune activation cascades, rather than directly with IDO enzyme, as often other immune-relevant molecules such as neopterin and IFN-γ are affected additionally. Also in this study, lavender oil treatment was able to reduce neopterin and IFN-γ levels in mitogen-treated PBMC.

Moreover, the increased production of neopterin during inflammation could also indirectly affect neurotransmitter concentrations. In human macrophages, neopterin is produced at the expense of tetrahydrobiopterin (BH_4_), an essential cofactor for several monooxygenases including tryptophan, phenylalanine and tyrosine hydroxylase [15]. Thus, beside serotonin synthesis, also catecholamine formation depends on BH_4_ availability. Interestingly, in depressed patients with a history of seasonal affective disorder, significantly lower plasma biopterin and tryptophan levels but elevated neopterin levels were found in comparison to healthy controls [41]. Thus, reduced BH_4_ levels in inflammatory conditions might negatively influence neurotransmitter production.

## Conclusion

We could show that lavender oil can suppress mitogen-induced tryptophan degradation and IFN-γ production *in vitro*, and influence on kynurenine and neopterin formation in activated as well as to a lower extent in unstimulated PBMC.

Also, the constituents (-)-linalool, (+)-α-pinene and (+)-limonene showed dose-dependent inhibitory effects on tryptophan breakdown in PHA-stimulated PBMC. Thus, the IDO suppressing activities of lavender oil might at least partially result from the concerted action of the analysed constituents, where also minor components may play an essential role.

The finding that lavender essential oil, a medicinal plant-derived natural multicomponent preparation, may be a source of pharmacological active substances that interfere with key immune activation cascades such as the IDO and GTP-CH-I pathway, is of central relevance for the understanding of its therapeutic efficacy.

However, as the reported effects might not reflect the whole activity spectrum of lavender oil. Further research is necessary to elucidate other neuro-immunological relevant activities and to confirm the *in vivo* relevance of our findings.
